# A Translator at Work: Samuel ben Judah of Marseilles’s Translation Notes on Alexander’s *De Anima*

**DOI:** 10.12688/openreseurope.20551.2

**Published:** 2026-03-09

**Authors:** Hanna Paulmann

**Affiliations:** 1 Religions, University of Hamburg, Hamburg, Hamburg, 20148, Germany

**Keywords:** Samuel ben Judah of Marseilles; Alexander of Aphrodisias; De Anima; Translation; Hebrew Philosophy; Hebrew Manuscripts; Medieval Philosophy; Aristotelian Psychology.

## Abstract

**Background:**

Samuel ben Judah of Marseilles’s mid-fourteenth-century Hebrew translation of Alexander of Aphrodisias’s *De anima*, made from Isḥaq ibn Ḥunayn’s ninth-century Arabic translation of the original Greek, survives in four manuscripts. Three of these contain Samuel’s notes on his translation, which disclose a wealth of information concerning textual problems he faced, editorial decisions he took, and his understanding of the text and its arguments.

**Methods:**

The present paper analyses these notes, which have never been studied before, according to philosophical and philological methods.

**Results:**

My analysis shows that Samuel is a philosophically engaged translator who approaches Alexander’s text with a text-critical attitude that enables him to render it in a more coherent and accessible form than that found in the Arabic manuscript that served as his source. Thus, this paper deepens our understanding of Samuel’s activity as a translator.

**Conclusions:**

These results shows that we should take translators seriously as philosophical agents in their own right and compels us to study manuscripts that contain translations to find and explore the translators’ own voices within or next to their works.

## 1. Introduction

Samuel ben Judah of Marseilles, a rather colourful figure of the intellectual circles of fourteenth-century France, translated Isḥaq ibn Ḥunain’s Arabic translation of Alexander of Aphrodisias’s *De Anima* in 1324 and then revised his translation in 1340. The work survives in four manuscripts, three of which contain his working notes covering textual problems, editorial decisions, and explanations as paracontent in the margins. These notes are an important first-person testimony of a scholar at work, and they can teach us both about his translating methods (including his editorial decision-making regarding variants that accompany the text) and about his understanding of the philosophical arguments in the text. Furthermore, some of Samuel’s notes refer directly to Isḥaq’s lost Arabic version of the work, which allows us a glimpse at the textual tradition of the Arabic text. Though previously recognized by scholars, the research on these notes so far has been very limited [ 1].

The present paper, written in the context of the HEPMASITE project that researches Jewish Philosophy on the manuscript level and as it was carried out by actual people in the real world, will study Samuel’s working notes on his translation, with a view to deepening our understanding of Samuel and his work as a translator. As such, it also serves as a contribution to the small but important body of scholarship about Hebrew translators’ working notes [ 2] and offers fundamental data for broader research in the fields of the history of Jewish philosophy and translation studies. In the course of this paper, it will become evident that Samuel is a philosophically engaged translator who approaches the text with a text-critical attitude.

To achieve this, I will first give a short introduction to Samuel and Alexander’s *De Anima* ( Section 2). Then, I will offer a detailed discussion of five of Samuel’s translation notes, which give insight into different aspects of his translation process ( Section 3). Finally, I will summarize the results of the discussion, contextualize them, and present the takeaways of this paper ( Section 4).

## 2. From Alexander to Samuel

Alexander (born between 140 and 165 CE in Aphrodisias, a city in Caria, modern-day Turkey) wrote many highly influential commentaries on Aristotle’s works and also composed original works in which he elaborates on and defends Aristotelian points of view. Aristotle’s doctrine of the soul served as material for Alexander on two separate occasions, but while most of his commentary on Aristotle’s *De Anima* was lost quite early ( 11, pp. 317–24), his independent work on the soul (together with its supplement, usually referred to as “Mantissa,” a collection of smaller texts on various matters) has survived, preserving access to his understanding of Aristotle's *De Anima* and rendering it a valuable resource for engagement with the history of Aristotelian psychology since the Middle Ages. For the most part, Alexander’s *De Anima* follows both the structure and the doctrine of Aristotle’s *De Anima.* However, he approaches the topic in a systematic way rather than making use of Aristotle’s dialectical manner and draws from the complete Aristotelian corpus to embed the discussion of the soul in its theoretical frame ( 12, pp. 1–3). With this approach, he not only clarifies Aristotle’s doctrine of the soul, but also presents it as a coherent part of Aristotle’s overall philosophical system. Alexander’s *De Anima* was translated into Arabic by Isḥaq ibn Ḥunain (d. 910 or 911 AD), who translated directly from the Greek ( 5, p. 317), but his translation is lost ( 2, p. xiv) [ 3].

Samuel completed his Hebrew translation of Alexander’s *De Anima* in 1324 in Murcia, southern Spain, and made it accessible to the public after a revision in Montélimar in 1340 under the title מאמר בנפש ( *Treatise on the Soul* ) ( 5, pp. 296, 298) [ 4]. As he was active in the first half of the fourteenth century, Samuel stands rather at the end of the translation movement that saw many philosophical works being translated from Arabic into Hebrew [ 5]. Samuel’s Hebrew translation survives in four manuscripts: Paris BN héb. 893, ^13^ Paris BN héb. 894, ^14^ Berlin Or. Oct. 332, ^15^ and, fragmentarily, in Munich Cod. Hebr. 389 ^16^ [ 6]. Of those four, no manuscript contains all the notes, and Berlin 332 contains none.

Samuel closes his translation with a colophon [ 7], in which he gives details of the translation process and methods. Many of the things he addressed in the colophon can be seen in the surviving manuscripts. He complains about the obscurity of the text, worsened by the chain of translations ( 5, p. 317), a remark that is also found in some of his translation notes in the margins of the text. Samuel also gives some details about his translation method. He states that in his first translation, he did not follow all the variant readings he found in the Arabic, but sometimes preferred the reading of the main text (p. 318). This can be seen in the manuscripts that contain variants in the margins that are marked with the Arabic letter *nun*, two of which Samuel discusses regarding which reading should be preferred. He describes that his guiding principle in editing was what he thought to be the truth (ibid.). Many of the things that he corrected during his revisions were amended according to his “conjecture and opinion and what seemed to me to be correct after extraordinary study” (p. 319). This too can be seen in the manuscripts, which contain marginal notes in which Samuel suggests or points out corrections. In the last part of his colophon, he mentions that he introduced divisions into the text according to its subject matter (pp. 319–20), which still exist in the surviving manuscripts.

## 3. Notes

In total, there are nineteen different notes written by Samuel found across the manuscripts and they include both philological and philosophical content. In the following discussion, I will examine five of them in detail to show Samuel’s engagement with the text during the translation process. All of the notes and their reference passages can be found alongside the Greek text and a corresponding English translation in the extended data ( 20, pp. 4–24). For simplicity’s sake, I have numbered them and will henceforth refer to them by their respective number.

### 3.1 Note 1: Engagement with a textual problem [ 8]

In his first surviving note, Samuel addresses a problem he has noticed in the text and offers alternative readings in an attempt to solve it. The passage in question belongs to the beginning of Alexander’s *De Anima*, in which Alexander discusses how matter and form constitute a body and in what way they contribute to the qualities of the body they constitute.

In the Greek passage (right half of Table 1a), Alexander argues that a compound of matter and form has its being in virtue of its form. His first premise is that every body, be it artificial or natural, is what it is in virtue of its form ( 2, 7.4–5). He then equates the form of a thing with its completion and perfection (Gr: τελειότης: *teleiotēs*; Hebr.: שלמות: *šelemut*) ( 2, 7.5–6) and draws the conclusion that “when converted” (Gr.: ἀντιστρέψαντι: *antistrepsanti*; Hebr.: כשהתהפך: *ke-še-hithapekh *), it is also true that every body has its being in virtue of its form and that this form is the perfection of its body ( 2, 7.4–8). The distinction that Alexander drew between artificial and natural bodies that is mentioned in this argument precedes this passage. Artificial bodies are those that are crafted by someone possessing an art, for example, a carpenter ( 2, 2.25–3.13). Natural bodies, on the other hand, are formed naturally without anybody intentionally crafting them, and are prior to artificial bodies ( 2, 3.13–17).

**Table 1a.** The reference text for note 1 in Hebrew and Greek with English translation.

Hebrew passage ( 13, f. 4א; 14, f. 3א; 15, f. 4ב; 16, f. 4א)	English translation	Greek passage ( 2, p. 7)	English translation ( 9, pp. 35–36)
[4א] הנה במחויב יאמר שכל אחד מן הגשמים הנה בצורה יהיה שלמותו אבל אם היה כל אחד מהגשמים אשר עמידתם במלאכה **ובעבורה יקראו** **הגשמים הטבעיים** הנה בצורה הוא מה הוא וזה הענין הוא שלמות על אחד מהם הנה זה האמר כשהתהפך היה אמת והוא שהדבר אשר בו כל אחד מהגשמים המורכבים מהיולי וצורה הוא מה הוא היה אותו הדבר הוא צורתו ושלמותו. במחוייב] במחויב: פ בצורה הוא מה] בצורה מה: ב על] כל: פ, ב, מ כשהתהפך] כשתהפך: פ הוא] הנה: ב היה] הנה: ב, מ	Therefore, it should necessarily be said that each body’s culmination is through a form. But if each body that is constituted by art, **and for its [i.e., art’s] sake, is called a natural body**, then it is what it is by form, and this [i.e., the form] is the culmination of each of them. Therefore, this statement, when converted, is true, and it is that the thing by which each body composed of matter and form is what it is the same thing, its form and culmination.	[7.2–8] […] εὐλόγως ἑκάστῳ τῶν σωμάτων ἐν τῷ εἴδει ἡ τελειότς ἂν εἶναι λέγοιτο. Ἀλλὰ εἰ κατὰ τὸ εἶδος ἑκάστῳ τῶν τε κατὰ τέχνην συνεστώτων **καὶ πολὺ πρότερον** **ἔτι τῶν κατὰ φύσιν** **τὸ εἶναι τοῦτο ὅ** **ἐστι**, καὶ τοῦτ΄ ἔστιν ἡ ἑκάστου τελειότης, καὶ ἀντιστρέψαντι ἀληθὲς τὸ καθ' ὃ τὸ εἶναί ἐστιν ἑκάστῳ τῶν ἐξ ὕλης τε καὶ εἴδος συνθέτων, τοῦτ’ εἶναι τὸ εἶδος αὐ τοῦ καὶ τὴν τελειότητα.	[…] then it would make sense to say that each body’s culmination is in its form. Yet, if each thing put together by art **and, ** **even prior to that, ** **each thing formed** **naturally** is just what it is in virtue of its form, and [the form] is the culmination of each, then it is true, when converted, that that in virtue of which every compound of matter and form has its being is its form and culmination.

**Table 1b.** Samuel’s note 1 in Hebrew and with English translation.

Note 1 in Hebrew ( 14, f. 3א; 16, f. 4א)	English translation
[3א] אמר שמואל המעתיק התיבות הארבעה הרשומות נ' לי שלא נבראו ואיני מתישב בשום פנים בהבנתם אך כן מצאתי וכן הנחתי ואפשר להעתיק תמורת תיבת **יקראו** תיבת יובאו או תיבת ישובו ולא אבין בשום פנים הארבעה] הארבע: מ נ’] נוח: מ יובאו] יוביו: מ	Samuel the translator said: It seems to me that the four words listed were not created [i.e., they were not in the original text], and I cannot make sense of them in any way, but so I found them and so I have left them. Instead of the word **יקראו**, it is possible to translate the word יובאו (“brought [forth]”) or the word ישובו (“become”). I do not understand [the text] in any manner [of reading it].

The Hebrew translation of the quoted passage (left half of Table 1a) differs from the sense of the Greek text as it is transmitted regarding Alexander’s antecedent, according to which every body, be it artificial or natural, is what it is in virtue of its form. Most notably, the Greek passage καὶ πολὺ πρότερον ἔτι τῶν κατὰ φύσιν ( *kai poly proteron eti tōn kata physin*: “and, even prior to that, each thing <formed> naturally”) undergoes a transformation in the corresponding Hebrew passage ובעבורה יקראו הגשמים הטבעיים ( *u-baʿavurah yiqqare’u ha-gešamim ha-ṭiveʿiyyim*: “and for its [i.e., art’s] sake, they are called natural bodies”). Instead of πολὺ πρότερον ( *poly proteron*: “prior”), the Hebrew text reads ובעבורה ( *u-baʿavurah:* “and for its sake”). Furthermore, in the Hebrew passage, a repetition of עמידתם ( *ʿamidatam*: “their constitution”) is neither assumed, as happens in the Greek with the word συνεστώτων ( *synestōtōn*: “put together”), nor explicitly repeated, but instead, the new verb יקראו ( *yiqqare’u*: “they are called” or “they are called upon”) is added. If יקראו is understood as “they are called,” then the Hebrew maintains that artificial bodies are called natural bodies, which differs from the Greek meaning, according to which natural bodies are prior to artificial bodies. This Hebrew reading renders the text philosophically problematic. Perhaps understanding יקראו as “they are called upon” makes better sense; it may mean that natural bodies are utilized for the sake of art, which is coherent with Alexander’s view. Nonetheless, with the Arabic translation lost, it remains unclear how exactly this phrasing came to be based on the Greek.

The second difference is the structure of the antecedent. In its Hebrew translation, the antecedent of the conditional in the Greek is broken up by an additional הנה ( *hinne*: “then”) to create a conditional within the antecedent. The new antecedent is “if each body that is constituted by art, and for its [i.e., art’s] sake, is called a natural body” and the consequent “then it is what it is by form.” This conditional, if true, would require a lot of additional information to explain why this should be the case.

Samuel’s note on this passage ( Table 1b) suggests that he noticed that the text is problematic in its current state. In his note, he addresses the four words ובעבורה יקראו הגשמים הטבעיים (“and for its [i.e., art’s] sake, they are called natural bodies”), suggesting that they were not in the original Greek text and noting that he cannot make sense of them. Still, he decided to leave them in the text and offers alternative translations for יקראו – namely, יובאו ( *yuve’u*: “they are brought forth”) and ישובו ( *yašuvu*: “they become, they return”) – in an attempt to solve it.

The verb יובאו has the basic meaning “to bring,” which can also take on a causal note of “to bring forth.” Replacing יקראו with יובאו would create a sentence that expresses that natural bodies are brought forth for the sake of art. Since natural bodies can, in fact, be used for art, this reading preserves the correct relation between natural and artificial bodies.

The second option Samuel mentions is ישובו, which in this case seems to carry the meaning “to become.” There are two ways to understand the passage with this meaning. Either it is read as artificial bodies becoming natural bodies, which would turn the actual relation between artificial and natural bodies upside down, or it is read as natural bodies coming to be for the sake of art, which is similar to the reading with יובאו.

All three options can be read in a way that is philosophically meaningful and that avoids identifying artificial and natural bodies. However, they do not give any more clarity to the first conditional that is formed from the antecedent in the Greek text by an additional הנה in the Hebrew text, so this passage remains problematic.

### 3.2. Note 6: Insertion of a correction into the text

In note 6, Samuel explains that he has inserted a negation into the text that he did not find in the Arabic manuscript that provided him with his source text. Even though he had no access to the Greek text, he correctly restored a negation that existed in the Greek, but was lost at some point during the transmission process, thereby remedying an absurdity created by the lost negation. It refers to a passage in which Alexander argues that the soul has more than one power.

Alexander’s argument is that if there was only one power of the soul, then the soul of every living being would be able to exert every kind of activity as long as adequate objects for this activity were present. There are, however, living beings that cannot exert some powers of the soul despite the presence of adequate objects. If only the lack of corresponding organs were to blame, then one would have to admit that these powers existed in vain in this living being, thus undermining the claim that nature does nothing in vain (right half of Table 2a). The Hebrew text follows the argument of the Greek text (left half of Table 2a).

**Table 2a.** The reference text for note 6 in Hebrew and Greek with English translation.

Hebrew passage ( 13, f. 13ב; 14, ff. 10ב–א; 15, f. 13ב; 16, f. 15א)	English translation	Greek passage ( 2, p. 27)	English translation ( 12, p. 53)
[13ב] וכבר אנו מוצאים קצת הב"ח לו מכחות הנפש ואי אפשר לו שיפעל שאר פעולותיה גם כן על שהענינים אשר יצטרך בם אל הפעולות אשר יפעל באותם הכחות נמצאים כי אומר אם אמר שאלו הפעולות **אין** אמנם יהיו בזה המין מן הב"ח לחסרון הכלים הנה כבר כפר שיהיה הטבע לא יפעל דבר בטל הב"ח] הבעל חיים: ב| הבעלי חיים: מ לו] לו כח: פ,ב,מ מכחות] מכוחות: ב ואי] אי: פ גם כן] ג"כ: פ בם] בהם: פ אל] אלו: פ באותם] באותן: ב אם] או: ב הב"ח] הבעל חיים: ב,מ	We find some animals that have a power of the soul, and it is impossible for them to perform its [i.e., the soul's] other activities, even though the things that it needs for the actions that it performs through the same powers exist. For if someone says that these actions will **not** be in these species of animals due to the lack of the [corresponding] organs, then he denies that nature does nothing in vain.	[27.9–13] […] εἶναι δὲ τινα <ζῷα>, ἃ δύναμιν ψυχῆς ἔχοντα μηκέτ΄ ἐνεργεῖν κατὰ τὰς ἄλλας ἐνεργείας δύναται καίτοι τῶν πραγμάτων ὄντων περὶ ἂ ἁι κατ΄ ἐκείνας τὰς δυνάμεις ἐνέργειαι. Εἰ γὰρ λέγοι τις δι΄ ὀργάνων ἀπορίαν ἐν τούτοις **μὴ** γίνεσθαι τὰς ἐνεργείας, ἀναιρήσει τὸ μηδὲν μάτην ποιεῖν τὴν φύσιν.	[…] and there are things that have a power of soul, but cannot engage in other activities beyond it, even when there are objects present which the activities corresponding to these powers concern. For if someone were to claim that these activities do **not** occur in them [solely] due to a lack of organs, he will undermine the claim that nature does nothing superfluous.

**Table 2b.** Samuel’s note 6 in Hebrew and with English translation.

Note 6 in Hebrew ( 13, f. 13ב; 14, f. 10א; 16, f. 15א)	English translation
[13ב] אמר שמואל המעתיק תיבת **אין** לא היתה בערבי שהעתקתי ממנו וחדשתי השלילה הזאת מדעתי בערבי] בערבית: פ ממנו] זה ממנו: פ, מ	Samuel the translator said: The word **“not”** was not in the Arabic from which I translated. I introduced this negation of my own accord.

In his note, Samuel describes how the negation אין ( *ein*) in the phrase “these actions will **not** be in these species of animals due to the lack of organs” was not in the Arabic manuscript from which he translated and that he added it ( Table 2b). Without this negation, this passage would be rendered absurd, since there is no meaningful way to say that an animal can exert certain activities because they lack the corresponding organ. Samuel’s intervention, therefore, eliminates the absurdity and corresponds to the text as it is transmitted in Greek.

It should be noted that changing the main text constitutes a rather drastic intervention. Samuel informs the reader about the change in his note, but notes are not always copied. A reader of the Berlin manuscript, ^15^ which does not contain Samuel’s notes, would not know that Samuel was the one inserting it. They would therefore be completely unaware that there was a problem in this passage and would moreover lack information to make up their own mind about which version is correct, which, in turn, affects the way the text is received and understood.

### 3.3. Note 2: Reproduction of a marginal note

Note 2 reproduces a marginal note that Samuel found in the Arabic manuscript from which he was translating, which explains the term ἐντελέχεια ( *entelecheia*: “completion”). He deems that it does not belong to the main text and attributes it to the Arabic translator Isḥaq ibn Ḥunain. However, a comparison with the Greek text as transmitted shows that parts of it belong to the main text, which gives rise to questions about the origin of this passage.

The passage to which this note refers is part of an argument that the soul is the first completion of a living being. In the Greek text, Alexander describes how Aristotle equates the culmination of a thing (Gr.: τελειότης: *teleiotēs*; Hebr.: שלימות: *šelemut*) with its completion. He explains that it is called “completion” because it is responsible for a thing becoming complete and that therefore, Aristotle characterized the soul as the “first completion” (ἐντελέχεια ἡ πρώτη: *entelecheia hē prōtē*) (right half of Table 3a).

**Table 3a.** The reference text for note 2 in Hebrew and Greek with English translation.

Hebrew passage ( 13, ff. ב8–9א; 14, f. 6ב; 15, f. 8א; 16, f. 9ב)	English translation	Greek passage ( 2, p. 16)	English translation ( 12, p. 43)
[8ב] וכבר התבאר שהצורה אשר היא למה שהיא לו צורה שלימות גם כן והיה ממנהג ארסטו' שיקרא השלמות ביוני **אנטלכיא** והשלמות הזה שני מינים אחד מהם קנין וכח והאחר הפעל אשר מאותו הכח והכח משני אלו הוא השלמות הראשון צורה] צורה הוא: פ שלימות] שלמות: פ, ב, מ גם כן] ג”כ: פ אנטלכיא] אנטלאכייה: פ| אנטלאכיא: ב|אנטאלכיא: מ	And it has already been explained that the form is a culmination for that of which it is a form. It is also in the habit of Aristotle to call the culmination **entelechy** in Greek. This completion is of two kinds. One of them is a disposition and potentiality, and the other is the actuality that [proceeds] from that potentiality. And the potentiality is the first completion of these two.	[16.4–10] […] τὸ δὲ εἶδος, οὗ ἐστιν εἶδος, ἐδείχθη καὶ τελειότης ὄν, ἔθος δὲ Ἀριστοτέλει τὴν τελειότητα καὶ **ἐντελέχειαν** λέγειν, ῶς τοῦ ἐν τῷ τέλει εἶναι τὸ πρᾶγμα οὗ ἐστιν οὖσαν αἰτίαν, εἰκότως αὐτῆς ἀπέδωκε τοιοῦτον τὸν λόγον: ἐντελέχεια ἡ πρώτη. Διττὴ γὰρ ἦν ἤ τελειότης, ἠ μὲν ἓξις τε καὶ δύναμις, ἡ δὲ ἀπο τῆς δυνάμεως ἐνέργεια, ὧν ἡ δύναμις ἦν πρώτη […].	[…] and it has further been shown that the form is a culmination of that of which it is a form; and it is characteristic for Aristotle to say that the culmination is a **completion, ** since it is responsible for the fact that the object to which it belongs is complete, it makes sense, then, that he offered this sort of characterisation: [the soul] is a “first completion.” For there are two kinds of culmination, (a) one which is a disposition and power and (b) another which is the activity issuing from that power, and of these the power is first […].

**Table 3b.** Samuel’s note 2 in Hebrew and with English translation.

Note 2 in Hebrew ( 13, f. 8ב; 14, f. 6ב; 16, f. 9ב)	English translation
[8ב] אמר שמואל המעתיק מצאתי בכאן הגהה אחת ומדעתי אינה מהשורש והיא זאת וענין זה השם בלשון יוני מה שיהיה בדבר כאשר תם ר"ל שזה הענין אמנם יהיה בסוף הענין אצל תמימות הדבר אשר הוא לו שלמות והיה זה השלמות סבה לפעל הנה במחויב הושם מאמרה והוא מליצה תכלול על הגדר ועל הרושם והיא גדרה **אנטלכיא** הראשון אמרתי הנה זאת ההגהה מדעתי היא לאסחק בן חנין המעתיק הראשון מהשורש] מהשרש: מ יוני] היוני: פ, מ לפעל] לפועל: פ במחויב] במחוייב: פ והוא] והיא: פ הרושם] הרשם: פ, מ והיא] והוא: פ אנטלכיא] אנטלאכייה: פ|אלאנטאכיא: מ אמרתי] אמרתי אני: פ חנין] חנן: מ	Samuel the translator said: I found one annotation here and I think that it is not part of the main text, and it is this: The meaning of this noun in Greek is “that which is in a thing when it ends,” meaning that in the end, this completion is what the thing has. That which is the completion for this thing is the cause for action. That which is the completion for this thing is the cause of action. Therefore, it is necessarily uttered in this way, and this is an expression that includes the definition and the description. And its definition is the first entelechy. I said: I think that this annotation was [made] by Isḥaq ibn Ḥunayn, the first translator.

The Hebrew text shows that the Arabic translation followed the Greek text closely, even transcribing ἐντελέχεια into Arabic, from which a Hebrew transcription was made (אנטלכיא), since the discussion is about a Greek term. However, the explanation of ἐντελέχεια and the characterization of the soul as a first completion (underlined in the Greek and in Caston’s translation in the table) is missing in the Hebrew main text. After equating the thing’s culmination with its completion, the text immediately continues by distinguishing between two kinds of culmination (right half of Table 3a).

In the note to this passage, Samuel explains that he found a note in the margins that does not seem to belong to the main text. He relates that note in Hebrew and, after the quotation, says that he thinks that it stems from the Arabic translator Isḥaq ibn Ḥunain ( Table 3b). Parts of the note he quoted contain the parts that are missing in the Hebrew main text: namely, the description of the meaning of the word ἐντελέχεια as “that which is in a thing when it ends” (מה שיהיה בדבר כאשר תם: *ma še-yihye be-davar ka-ašer tam*). After that, however, there is an additional follow-up explanation that does not exist in the Greek, which describes that the completion is what a thing has in the end and that this completion is the cause of action. The Greek phrase “it makes sense, then, that he offered this sort of the characterisation” is rendered as “Therefore, it is necessarily uttered in this way, and this is an expression that includes the definition and the description,” and the quotation ends by saying that “its” definition, which, according to context, should be the soul’s definition, is a first completion ( Table 3b).

The note that Samuel quotes is an explanatory passage that is missing from the Hebrew main text, but contains an additional explanation that is not found in the Greek. This constellation raises questions about the origin of this passage and the additional explanation within it. Bruns does not note that this passage is missing in his sources in his critical apparatus. Nevertheless, it remains an open question whether it was removed in the Arabic tradition or in the Greek tradition, and also, if it was removed in the Greek tradition, whether it was originally part of the main text or not. The same applies to the question of where the additional explanation comes from and how it entered the passage in question. This note, therefore, tells of a problem that might go back to the Greek tradition, and as such, it is also of interest to scholars of classics as it opens the question whether (parts of ) the passage missing in the Hebrew main text was originally composed by Alexander or not and how this text was transmitted in the Greek context.

### 3.4. Note 3: Criticism of an argument

In note 3, we find a note by Samuel that does not pertain to the translation. Rather, it contains a critical stance towards Alexander’s manner of argumentation.

This passage is part of Alexander’s argument that forms cannot be bodily. After giving an argument as to why forms are not bodily according to his own conception of a body, he also argues that forms are not bodily according to the Stoic conception of a body either, even though qualities – and with that, forms – are bodies according to the Stoics ( 12, p. 100n162), thus showing an inconsistency in the Stoic doctrine of bodies. The overall claim for which Alexander wants to argue is that forms cannot be bodies (even according to the Stoics), and he does so by way of a *reductio* argument. According to the Stoic conception of a body, he says, a body is either matter or composed of matter. In the passage quoted in the table, he denies that form can be matter, because matter does not possess any quality, while form is, in a way, a quality (right half of Table 4a). After this passage, Alexander continues by giving several arguments that the form cannot be composed of matter and form either, thus concluding that the form cannot be bodily according to the Stoic definition of a body ( 2, 17.18–18.10).

**Table 4a.** The reference text for note 3 in Hebrew and Greek with English translation.

Hebrew passage ( 13, f. 9ב; 14, f. 7א; 15, f. 9ב; 16, f. 10א)	English translation	Greek passage ( 2, p. 17)	English translation ( 12, p. 44)
[9ב] ומפני זה גם כן אינה גשם לפי שאי אפשר ולא כפי מאמר מי שיאמר בשהיא גשם פשוט או היולי או מהיולי וצורה כמו שיחשבו בעלי החשך **שתהיה הצורה גשם** וזה שהצורה אינה היולי לפי שההיולי בלתי מתאייך ואולם הצורה היא איכות מה גם כן] ג"כ: פ מתאייך] מתאיך: ב, מ	And because of this, it [i.e., a form] is also not a body, since this is not possible; neither according to whoever says that it [i.e., the form] is a simple body, or matter, nor [a composite of] matter and form, as the followers of [the way of] darkness [i.e., the Stoics] [ 9] thought, **that the form is a body**. For the form is not matter, because matter does not receive qualities, whereas the form is a certain quality.	[17.15–18] Ἀλλ΄ οὐδὲ κατὰ τοὺς λέγοντας πᾶν σῶμα ἢ ὕλην ἢ ἐξ ὕλης, εἶναι (ὡς τοῖς ἀπὸ τῆς Στοᾶς δοκεῖ) **εἴη ἂν τὸ εἶδος** **σῶμα**. Οὔτε γὰρ ὕλη τὸ εἶδος (ἡ μὲν γὰρ ἄποιος, τὸ δὲ ποιότης τις)	**A form, furthermore, ** **cannot be a body** along the lines of those who claim, like the Stoics, that every body is either matter or composed from matter. For form is not matter, since the latter is qualityless, while the former is a kind of quality.

**Table 4b.** Samuel’s note 3 in Hebrew and with English translation.

Note 3 in Hebrew ( 13, f. 9ב; 14, f. 7א)	English translation
[9ב1] אמר שמואל המעתיק אחר אמרו **שתהיה הצורה** **גשם** היה ראוי שיביא מופת מבאר הדרוש ההוא שהצורה אינה גשם ואם הוא מבואר בעצמו היה ראוי שיאמר אחריו והצורה אינה היולי ההוא] ההוא והוא: פ	Samuel the translator said: After he said **that** **the form is a body, ** he should have brought a demonstration explaining this desideratum that the form is not a body. Even if it is self-explanatory, he should have continued [by saying] “and the form is not matter.”

The Hebrew diverges from the Greek in two significant aspects. The first is that the enumeration of options as to what a body might be in the Greek text no longer applies to bodies, but to the form. In the phrase “whoever says that it is …” that precedes the enumeration, the “it” is a feminine pronoun (בשהיא: *be-še-hi’*) and thus refers to the feminine noun “form” (צורה: *ṣurah*) and not to “body” (גשם: *gešem*), which is masculine (left half of Table 4a).

The second difference is that simple bodies (גשם פשוט: *gešem pašuṭ*) were added to this enumeration as a first option (left half of Table 4a). This addition can be explained by Alexander’s discussion that the soul cannot be a simple body, which follows his discussion of whether forms can be composed of matter and form ( 2, 19.21–20.26).

Bruns’s critical apparatus for the Greek text does not mention this variant. However, it is possible either that the simple bodies were an original part of the enumeration, but are missing from the Greek manuscripts that survived – they might have been removed because simple bodies are themselves composed of matter and form and therefore seemed redundant – or that they were added in some manuscripts to make the list of options at the beginning correspond with all the discussed options of how a form (or the soul, which is a kind of form) could be a body.

These two changes create a text stating that forms cannot be bodies, not even according to those who think that forms are either simple bodies or matter or composed of matter and form. So, while the conclusion of the argument is still the same – namely, that forms are not bodies – the *reductio* argument does not work as it does in the Greek text, because now, instead of enumerating what kinds of being count as a body, the Hebrew text enumerates what kinds of beings count as a form. In the passage in the table, Alexander rules out that forms are matter, and afterwards, he argues that forms are not composed of matter and form either ( 2, 17.17–19.20), nor are souls simple bodies ( 2, 19.21–20.26). So if, according to the Hebrew text, the Stoics claim that forms are either simple bodies or matter or composed of matter and form, and the only thing for which Alexander subsequently argues is that forms are neither, then the conclusion that can be drawn from this is that the Stoics had a wrong conception of form, not that forms are not bodies. Therefore, there is no explicit argument in the Hebrew text for the claim that forms are not bodies.

Samuel addresses this problem in his note. He complains that while Alexander wants to argue against the claim that a form is a body, he ends up providing an argument that form is not matter (and later that form is not composed and that souls are not simple bodies), which only addresses the Stoic conception of form, not the claim that forms are not bodies. This discrepancy leaves Samuel demanding that Alexander close the gap with a proof for the original claim that the form is not a body. Furthermore, even if such a claim were self-explanatory, he suggests a different continuation of the sentence. In the main text, the sentence continues with “ *for* form is not matter” (וזה שהצורה אינה היולי: *we-ze še-ha-ṣurah einah hiyuli*). This phrasing suggests that the argument that forms are not matter has an explanatory function in the argument that forms are not bodies, which, as we have seen, does not work in the Hebrew text. Instead, Samuel suggests changing it to “ *and* form is not matter” (והצורה אינה היולי: *we-ha-ṣurah einah hiyuli*) ( Table 4b). This phrasing detaches the argument that form is not matter from the claim that form is not a body and instead starts a new argument, thus doing justice to the altered context of the argument.

### 3.5. Note 7: Engagement with a textual variant

The last note I will discuss is note 7, in which Samuel discusses a variant found in the margins. This variant belongs to a group of marginal notes containing variants that are marked with the Arabic letter *nun.* In his colophon, Samuel mentions that he found marginal notes in the Arabic manuscript from which he was working and that he did not always follow them, depending on whether the marginal note or the main text seemed more reliable to him ( 5, p. 318). Note 7 attests to this engagement with variants in the margins to which Samuel refers.

The passage to which note 7 refers discusses the relationship between being nourished and growing.

In the Greek passage, Alexander explains why nourishing and growing are not the same activity. Animals nourish themselves during their whole lives, which makes nourishment the most continuous (συνεχεστάτη: *synechestatē*) activity of the soul. Growing, on the other hand, is something animals only do for a certain period of their lives, since some start to shrink after reaching a certain age (right half of Table 5a).

**Table 5a.** The reference text for note 7 in Hebrew and Greek with English translation.

Hebrew passage ( 13, f. 18א; 14, f. 13ב; 15, ff. 17ב–א; 16, f. 19ב)	English translation	Greek passage ( 2, p. 35)	English translation ( 12, p. 61)
[18א] וזה שהבעל חיים מה שהתמיד נשאר הנה הוא יזון ולכן היה זה הפעל **למטה** **מפעולות [נ' בלעדי** **פעולות]** הנפש ואין מה שיזון הנה הוא לעולם יצמח אחר שהיה מה שיזון הוא יזון מה שהתמיד נשאר והיה מה שכבר בלה וחסר הנה הוא נשאר שהבעל חיים] שהב"ח: פ	For as long as an animal endures, it is nourished. Thus, this activity is **below** **the activities [variant: is** **without the activities]** of the soul. That which is nourished will not always grow, because that which is nourished is nourished as long as it endures and that which is already decaying and decreasing [still] endures.	[35.10–13] Τρέφεται μὲν γὰρ ἀεὶ τὸ ζῳον, ἔστ΄ ἄν ᾖ (διὸ καὶ **συνεχεστάτη** ἥδε **τῶν** ψθχικῶν **ἐνεργειῶν**), αὔξεται δὲ οὐκ ἀεὶ τρεφόμενα, εἴ γε τρέφεται μὲν ἔστ΄ ἂν ᾖ, ἔστι δὲ καὶ τὰ γηράσκονά τε καὶ μειούμενα.	For an animal is always being nourished for as long as it exists – it is for just this reason the **most continuous of** soul **activities**. But things which are nourished are not always growing, since they are nourished as long as they exist, but some also shrink as they age.

**Table 5b.** Samuel’s note 7 in Hebrew and with English translation.

Note 7 in Hebrew ( 13, f. 18א; 14, f. 13ב; 16, f. 19ב)	English translation
[18א1] אמר שמואל המעתיק הנסחא הפנימית עקר והחצונה אחשוב שהיא מוטעית אמר] ואמר: פ והחיצונה] והחצונה: פ, מ מוטעית] מוטעת: מ	Samuel the translator said: The inner formulation [i.e., the one in the main text] is the main one and I think the outer one [i.e., the one in the margins] is wrong.

The Hebrew text contains the same kind of explanation. An animal is nourished as long as it “endures” (מה שהתמיד נשאר: *ma še-hitmid niš’ar*), meaning as long as it exists. Therefore, as per the main text, it is “below” (למטה: *le-maṭa*) the activities of the soul. This notion diverges from the Greek, which describes this activity as “continuous.” The final part of the explanation concerns the contrast between growing and nourishing by saying that things will not always grow, even though they are nourished, because they are nourished as long as they exist, but things that are already decreasing, and thus no longer growing, still exist (left half of Table 5a).

In his note, Samuel discusses a marginal note he found, writing that the “inner formulation” (הנסחא הנסחא הפנימית: *ha-nusḥa’ ha-penimit*), meaning the formulation in the main text, is correct and that he thinks that the “outer one” (החצונה: *ha-ḥiṣona*), meaning the one in the margins, is wrong. The formulation this note addresses is “below the activities” in the main text and “without the activities” (בלעדי פעולות: *bil‘adei pe‘ulot*) in the margins. Samuel expresses his preference for nourishing being understood as an activity “below” the activities of the soul ( Table 5b).

The formulation in the margins heavily contradicts the Aristotelian definition of the soul as a life principle that enables living beings to perform the activities that are typical of living beings. Nourishing is an activity shared by all living things and as such, it cannot be “without the activities of the soul”; it is necessarily an activity of the soul and is treated as such by Alexander. Therefore, Samuel gives preference to the formulation in the main text that nourishing is an activity “below the activities of the soul.” What this means exactly, and how the Greek word συνεχεστάτη became למטה in the Hebrew, remains unclear.

## 4. Conclusion

In this paper, I have analysed Samuel ben Judah of Marseilles’s translation notes on his translation of Alexander of Aphrodisias’s *De Anima.* My analysis shows that Samuel followed the argument of the text in an engaged manner and with a text-critical attitude. His reference in this process is always Alexander’s doctrine, which is reflected in his editorial decisions and solutions.

Samuel’s active engagement enabled him to notice passages where the text seems wrong, incomplete, or obscure. In his notes, he displays an awareness of these problems and attempts to solve them via explanations or corrections, even going so far as to express his critical stance towards Alexander, as we saw in the discussion of note 3. A comparison with the Greek text as it is transmitted reveals that almost all of the corrections he suggests or implements correspond to the Greek text. The two exceptions to this are two corrections we have not discussed here, but which can be found in the extended data for this article. ^20^ One is mentioned in note 17, which does not have a direct equivalent in the Greek, but is philosophically justified ( 20, pp. 20–21). Another is suggested in note 4, which changes the text compared to the Greek, but creates a less problematic text on a grammatical level and an argument that still serves to support Alexander’s position ( 20, pp. 8–9). The passages Samuel deemed obscure or incomplete also differ from the Greek text, either in wording or in sentence structure. As such, they bear witness to textual problems that might go all the way back to the Greek tradition, as discussed in the context of note 2.

Furthermore, Samuel did not only rely on the main text for his translation, but also took into account marginal notes that he found in the Arabic manuscript. In his colophon, he mentioned the existence of marginal notes containing variants in the Arabic manuscript from which he was translating. As we have seen, note 7 showcases his engagement with these marginal notes, which belong to a group of marginal notes marked with the Arabic letter *nun.* This allows us to trace this note – and potentially the whole group of notes – back to the translation process itself. The same holds for note 15, which we have not explicitly discussed here.

Samuel’s translation notes, therefore, demonstrate how he tried – often successfully – to illuminate obscure passages of the text and to amend errors from the Arabic manuscript he was working with without any access to the Greek source, thus introducing Alexander’s text to the Hebrew philosophical audience in a more coherent and more accessible rendering.

Overall, Samuel’s notes also show his personality as we know it from his biography and the colophons. He was able to translate Alexander’s *De Anima* in this engaged manner thanks to his philosophical education. He expresses his opinions very directly and does not shy away from using his own judgement to make amendments to the text, which enforces the impression that he had a high opinion of his own skills. Something we need to keep in mind while thinking about his capabilities or his achievements is that Samuel was active during a rather late phase of the philosophical translation movement. At that time, technical terms had already been coined and, to a certain degree, standardized, there was a consolidated corpus of scientific works in Hebrew translation, and the act of translating had become professionalized. Therefore, he was in a privileged position regarding his translation activities.

To conclude, this case study on Samuel’s notes on his translation of Alexander’s *De Anima* gives an insight into the influence that translators’ editorial choices can exert regarding how the target audience might perceive the text. In this case, Samuel’s influence on the audience is even twofold: On the one hand, he interferes with the main text itself and on the other hand, the notes – being paracontent – are the “threshold” ( 23, p. 142) that shapes the way in which the audience understands the text by sharing his opinion on various passages. The text-critical notes seem to play a special role, since they do not only serve as a “threshold” to the content of the text, but also create an awareness of these textual problems and enables the audience to understand Alexander’s *De Anima* as the product of its transmission history from the Greek, over the now lost Arabic into Hebrew. At the same time, these notes portray Samuel’s agency to take relevant decisions in his translation process (cf. 24, p. 89) and are a very explicit expression of the translator’s voice, which is difficult to discern just by its “discursive presence in a text” ( 25, p. 5).

The main takeaway from that is that we should upgrade our notion of translators and take them seriously as engaged, philosophical agents in their own right. Therefore, we should study the manuscripts that contain translations with their paracontent to find and explore the translators’ own voices within or next to their works. This applies not only to other translators, but also to other translations by Samuel: for example, his translations of Averroes’s Middle Commentary on Plato’s *Republic* and Averroes’s Middle Commentary on Aristotle’s *Nicomachean Ethics*, which are the only other two translations he revised aside from his translation of *De Anima* ( 6, pp. 24–26). Studying these translations will help us to deepen our understanding of his activity as a translator. Moreover, we can conduct comparative studies of Samuel and the other translators of the translation movement such as Samuel ibn Tibbon or Moses ibn Tibbon on the basis of case studies dedicated to their approach to translating. Since Samuel was a rather late translator, a comparison with earlier translators might allow us to obtain a more detailed understanding of how techniques and the approach to translating changed during the translation movement. Further, these notes in particular and paracontent in scientific medieval manuscripts in general is not only interesting to scholars of medieval philosophy. It also offers an enriching perspective to the research on paracontent and translator’s (foot-)notes in translation studies, which, so far, has been primarily concerned with modern occurrences of footnotes in translations of printed works, where especially the text-critical aspect of paracontent plays a minor role and, therefore, did not receive much attention.

### Ethics & consent

Ethical approval and consent were not required.

## Data Availability

The data for this article consists of manuscripts, which are available online as follows (cf. Reference section): Paris, Bibliothèque nationale de France, hébreu 893. Accessed February 27, 2025. https://gallica.bnf.fr/ark:/12148/btv1b10546745w. Paris, Bibliothèque nationale de France, hébreu 894. Accessed February 27, 2025. https://gallica.bnf.fr/ark:/12148/btv1b10546746b. Berlin, Staatsbibliothek, Or. Oct. 332. Accessed February 27, 2025. http://resolver.staatsbibliothek-berlin.de/SBB0001D5E300000000. Munich, Bayrische Staatsbibliothek, Cod. Hebr. 389. Accessed February 27, 2025. https://mdz-nbn-resolving.de/urn:nbn:de:bvb:12-bsb00103865-4. The extended data for this paper include a brief description of these codices as well as the tables offering the edition and English translation of Samuel’s notes, accompanied by relevant portions of Alexander’s text. They are available under the terms of the Creative Commons Attribution 4.0 International license (CC-BY 4.0) in: OSF: A Translator at Work: Samuel ben Judah of Marseilles’s Translation Notes onAlexander’s De Anima - Extended Data. https://doi.org/10.17605/OSF.IO/XRYD6. ^20^ The following extended data belong to this project: version 2_Samuel’s Translation Notes_Appendices.pdf License: CC-By Attribution 4.0 International

## Footnotes

^1^
Research on Samuel’s translations and his notes can be found in Günsz, ^1^ which describes the Hebrew manuscripts and printed the section of Samuel’s translation that deals with the intellect as well as part of the colophon; Alexander of Aphrodisias, ^2^ which contains parts of Steinschneider’s translations from the Hebrew text, his comments about some passages, and even some of Samuel’s translator’s notes; Berliner and Hoffmann, ^3^ in which Steinschneider printed the complete colophon (pp. 8–10); Steinschneider, ^4^ which gives a brief overview of Samuel’s translation and the manuscripts in which it is preserved (pp. 152–53); and, more recently, Berman, ^5^ which gives an account of Samuel’s life and works and contains a translation of his colophons, and finally, in Berman’s editions of two of Samel’s translations ( 6 and 7).

^2^
Namely, Fontaine, ^8^ which discusses ibn Tibbon’s translation notes; Fraenkel, ^9^ which studies ibn Tibbon’s glosses and analyses them in order to learn about his translation methods, his way of explaining technical terminology, and his understanding of Maimonides’s text; and Meyrav, ^10^ which is dedicated to Moses ibn Tibbon’s working notes and retraces the history of translation and revision of the Hebrew version of Averroes’s Epitome of the *Physics.*

^3^
The Greek text also served as the source for Latin translations, which have no relevance for this paper, since, being of a later date, they did not influence the Hebrew translation. Hence, I will merely note briefly that there is one translation by Hieronymus Donatus that was published in 1495 and a partial translation dated to the sixteenth century by a certain Antonius de Albertis, who may, according to F. Edward Cranz, be identical with Antonio di Niccolò degli Alberti (born 1495) ( 17, pp. 84–88; 2, p. xiv).

^4^
For the most recent efforts to illuminate Samuel’s biography and works, see Berman, ^5^ ^–^ ^7^ and Zonta. ^18^

^5^
For information on the timeline of this translation movement and the different ways philosophy became accessible in Hebrew, see Harvey. ^19^

^6^
I have given a more detailed description of the manuscripts in the extended data ( 20, pp. 1–4).

^7^
The abridged version of Samuel’s colophon to his translation of *De Anima* found in Berlin 332 was first published by Günsz. ^1^ The first complete printing of the colophon as found in the two Parisian manuscripts, which was undertaken by Steinschneider, can be found in Berliner and Hoffmann ( 3, pp. 8–10). It was reprinted in the chapter on Samuel in Renan ( 21, pp. 553–67) and translated in its entirety by Berman. ^5^

^8^
This note is the first one in Paris 894. Despite the four words under discussion being marked by two dots on top of each in Paris 893 (which is the marking used for all the words discussed in Samuel’s notes in this manuscript), the note itself is not found in Paris 893 and is thus the only surviving note that is not found in that manuscript.

^9^
It is not clear how exactly the Stoics became “followers of the way of darkness.” In Bruns’s edition, Steinschneider remarks that the Arabic term might have been *Ahl al-ruwāq*, which Samuel might not have understood ( 2, p. 17). Herbert A. Davidson, on the other hand, mentions that the Arabic translation of Alexander’s *De Intellectu* translates the Stoics as “members of the shaded area” ( *al-miẓalla*) ( 22, pp. 297–80), which might serve as an alternative explanation for Samuel’s translation of Stoics as “followers of the way of darkness.” There is only one other occurrence of the Stoics in Alexander’s *De Anima* ( 2, 26.16), which Samuel translated in the same way as he did in this passage ( 13, f. 13r).
