# Supplementary material for: A Translator at Work: Samuel ben Judah of Marseilles’s Translation Notes on Alexander’s De Anima
Source: Open Res Eur. 2026 Mar 9;5:306. Originally published 2025 Oct 7. [Version 2] doi: 10.12688/openreseurope.20551.2 (PMC12639282; doi:10.12688/openreseurope.20551.2)
Supplement: Peer review history

# Reviewer report: Reviewer response for version 1

**DOI:** 10.21956/openreseurope.22236.r62588

**Published:** 2026-01-22

**Authors:** Michael Engel

**Affiliations:** 1 Hochschule für Jüdische Studien Heidelberg, Heidelberg, Germany

This is an important contribution to the field, both regarding Alexander´s translations into Hebrew but also to the general study of Alexander from Aphrodisias. 

 Some comments:

 a) p. 3: Author writes: According to Moritz Steinschneider, Samuel’s translation is based on a good Arabic

 manuscript (4, p. 152), and he closely follows the Arabic text (2, pp. xiv–xv). As it stands this assertion feels a bit out of context. Does the author agree or disagree, and is she to refer to this later? 

 b) p. 3 fn. 3: if the Latin has no relevance, which certainly may be the case, I would briefly explain why. 

 c) p. 4 n.8: author writes: “Despite the passage being marked, it is not found in Paris 893 and is thus the only surviving note that is not found in that manuscript.” This is not entirely clear, maeked as what? And why is the marking relevant to what follows in the sentence? I would consider repharasing.

 d) p. 6 3.3. “Note 2: Reproduction of a marginal note” I would add “from the Arabic.”

 e) p. 7, author writes: “he also argues that forms are not bodily according to the Stoic conception of a body, because according to the Stoics, … forms are bodies.” This is a bit confusing

 f) Could it be that in note 7, למטה מכוחות הנפש means something like „the lowest among the faculties of the soul“? 

 g) p. 10, author writes: “ In his notes, he creates an awareness of these problems” perhaps “shows” or “displays” instead of “creates”? 

 h) pp. 10-11 to me it was a bit unclear what the author means by “extended data for this article”

If any, are all the source data and materials underlying the results available?

Yes

Is the work original in terms of material and argument?

Yes

Is the work clearly and cogently presented?

Yes

Is the argument persuasive and supported by evidence?

Yes

Does the research article contribute to the cultural, historical, social understanding of the field?

Yes

Does it sufficiently engage with relevant methodologies and secondary literature on the topic?

Yes

Reviewer Expertise:

Medieval Jewish and Latin Philosophy

I confirm that I have read this submission and believe that I have an appropriate level of expertise to confirm that it is of an acceptable scientific standard.

# Response

**Published:** 2026-02-05

**Authors:** Hanna Paulmann

**Affiliations:** Religions, University of Hamburg Faculty of Humanities, Hamburg, Hamburg, Germany

Dear Prof. Dr. Michael Engel,   thank you very much for taking the time and effort to review this paper and for your thoughtful suggestions. When I wrote this paper, I wanted to compile all the information available. However, you are right that (a) the statement by Steinschneider that the Arabic manuscript was good or that Samuel followed it closely it is out of context, as I do not refer to this later. Steinschneider does not give any explanations or arguments for his statement, and since the Arabic translation is lost, there seems to be no way to proof it. Therefore, I have removed this sentence. Further, I have (b) added an explanation why the Latin translations are not relevant for the paper, (c) rephrased note 8, and (e) tried to clarify the confusing passage about the Stoic conception of bodies. The (d) addition of “from the Arabic” to the title of section 3.3 would suggest that this marginal note originated in the Arabic, but since it is not certain where and when this marginal note came to be, I would prefer to refrain from this addition. Regarding f) the translation of למטה מכוחות הנפש as „the lowest among the faculties of the soul“, this understanding is definitely something Alexander would subscribe to, but even if למטה could be understood as “lowest”, it still remains unclear how συνεχεστάτη became למטה in the Hebrew. According to your suggestion, I have (g) replaced “creates” with “displays”. Since this new version emphasises the influence on the audience according to the function of paracontent as a “threshold” to the text, the phrasing that he “creates an awareness” now appears later in the conclusion. The (h) “extended data” refers to my edition and translation of all of Samuel’s notes together with their respective passages of Alexander’s *De Anima *in Greek and in Samuel’s Hebrew translation. According to the paper guidelines, this kind of material is not published together with the paper, but is uploaded to a separate data repository. The link to the extended data can be found under [20] in the reference list.   Best regards, Hanna Paulmann

# Reviewer report: Reviewer response for version 1

**DOI:** 10.21956/openreseurope.22236.r62584

**Published:** 2025-11-24

**Authors:** Daria Vasyutinsky Shapira

**Affiliations:** 1 Tel Aviv University, Tel Aviv, Israel

This publication discusses the notes left by Samuel ben Judah of Marseilles on his Hebrew translation of the Arabic version of Alexander of Aphrodisias’s *De anima*. The author analyses the notes and reaches the conclusion that the translation was not mechanical but critical, and that Samuel even corrected some mistakes that derived from the Arabic translator's misunderstanding of the Greek original (which Samuel didn’t have). The research is based on four surviving manuscripts of Samuel ben Judah of Marseilles’s work and secondary sources. In addition, the extended data is provided separately, which includes the detailed description of the four manuscripts and all of Samuel’s notes. This is a solid and high-quality piece of research that makes a significant contribution to its field.

 Suggested improvements:

 The diacritics in the abstract should be fixed.

 The Hebrew texts in the tables are scans from a printed book; however, the references are to the manuscripts. Moreover, in some tables (1b, 2b, 4b, 5b), the scanned text is of such low resolution that it becomes unreadable.

 It would be helpful to provide the text of the colophons, at least partly. They are mentioned many times; it makes sense to quote them.

 In the extended data, I suggest replacing the 19th-century paleographical descriptions with modern ones or removing them altogether. BnF hébreu 893 is written, in today’s terminology, in Sephardic cursive. Different hands indeed appear, but not on 23v (this information is, anyway, irrelevant for this research), but on pages 1r and 1v. In BnF hébreu 894, the first work in the compilation is in Sephardic cursive, and the second one (48r and further) in Sephardic semi-cursive. I would also recommend extreme caution when dating based on these script types.

If any, are all the source data and materials underlying the results available?

Yes

Is the work original in terms of material and argument?

Yes

Is the work clearly and cogently presented?

Yes

Is the argument persuasive and supported by evidence?

Yes

Does the research article contribute to the cultural, historical, social understanding of the field?

Yes

Does it sufficiently engage with relevant methodologies and secondary literature on the topic?

Yes

Reviewer Expertise:

Medieval Hebrew paleography and codicology; digital Hebrew paleography.

I confirm that I have read this submission and believe that I have an appropriate level of expertise to confirm that it is of an acceptable scientific standard.

# Response

**Published:** 2026-02-05

**Authors:** Hanna Paulmann

**Affiliations:** Religions, University of Hamburg Faculty of Humanities, Hamburg, Hamburg, Germany

Dear Dr. Daria Vasyutinsky Shapira,   thank you very much for taking the time and effort to review this paper and for your thoughtful suggestions. The diacritics were represented correctly in my manuscript and the proof and I do not know why they were not correctly presented in the published version of this paper. I have fixed them in the new version, but might have to contact the publisher after publication, if that does not solve the problem. The same holds for the Hebrew texts in the tables. They are not scans from a printed book, but my own transcription of the passages as they appear in the manuscripts (which is why the reference is to the manuscripts), and they are represented in a text format in my manuscript and the proof. I do not know why they appear in a picture format ­– a low resolution picture at that – in the published version. That too, I fixed in my new version, replacing the pictures with the original text, and I will contact the publisher if this problem will reappear after publishing. Regarding your suggestion of providing (parts of) the colophon (the one to *De Anima* in the two Paris manuscripts is the one I refer to), I agree that it might be helpful, and if it had not already been published in Hebrew (Steinschneider 1887 pp. 8–10, reprinted in Renan 1893, pp. 565–66), and in an English translation (Berman 1967, pp.317–320), I would have added it to the extended data. Since it is, however, published, I prefer to refer to the respective places where it can be found. Finally, I have changed the palaeographical terminology from “Provençal” to “Sephardic” according to your suggestion. Since the different hands are indeed irrelevant for my argument, I have removed this information from the description. In general, the description of the manuscripts is supposed to supplement the paper by giving more information on the manuscript to those that are interested, but it does not aim for completeness or precision. The dating of the manuscripts in my description, therefore, is derived from other sources that describe the respective manuscripts and is not supposed to be the last words on that matter.   Best regards, Hanna Paulmann

# Reviewer report: Reviewer response for version 1

**DOI:** 10.21956/openreseurope.22236.r62083

**Published:** 2025-11-21

**Authors:** Richard Pleijel

**Affiliations:** 1 Stockholm University, Stockholm, Sweden

This article studies the notes of the translator Samuel ben Judah of Marseille in his translation of the Greek philosopher Alexander of Aphrodisias's treatise *De anima*. The translation was made from an Arabic translation (the manuscript now lost) into Hebrew. The author studies a selection of Samuel's notes and compares the main body of the Hebrew text with the Greek source text of the original work. Special consideration is taken to Samuel's notes of which English translations are given. The results show that Samuel was an active translator whose "voice" can be discerned through the notes. Samuel took an active part in the translation as he made his own editorial and text-critical decisions, revising or amending the source text where he considered this necessary.

 The article is well-written. The data is presented in a clear way and the arguments are easy to follow. The presentation of the textual material in the different tables is accurate, with the exception of Table 4a where text from the discussion (main text) seems to have mistakenly been inserted in the English translation in the second column.

 While the article is well-written and the material cogently discussed, however, there could have been more engagement with research from relevant fields so as to contextualize the discussion more. At present, the article is very narrowly focused on Samuel and his notes. What I found was lacking was the greater implications for fields such as textual criticism and translation studies. The author states in the conclusion (p. 10) that "Samuel was not translating mechanically," but who would argue that translators *do *translate "mechanically"? From the perspective of translation studies, such a claim was refuted several decades ago. The author could also have benefited from translation studies research on translator notes, specifically as way of discerning translators' "voices" (e.g., Outi Paloposki, "The translator's footprints," 2010, or Cecilia Alvstad and Alexandra Assis Rosa, "Voice in retranslation," 2015).

 The author states on on p. 7 that the note discussed in Table 3a "tells of a problem that might go back to the Greek tradition, and as such, it is also of interest to scholars of classics." This could also have been expanded on to show the implications for the field of classics. Finally, another context I found lacking is the larger one of the project which the present article apparently is a part of. This could have helped the reader to understand more precisely *why *it is of interest to study the notes of Samuel, beyond this particular translator and his texts.

If any, are all the source data and materials underlying the results available?

Yes

Is the work original in terms of material and argument?

Yes

Is the work clearly and cogently presented?

Yes

Is the argument persuasive and supported by evidence?

Yes

Does the research article contribute to the cultural, historical, social understanding of the field?

Partly

Does it sufficiently engage with relevant methodologies and secondary literature on the topic?

Partly

Reviewer Expertise:

Translation studies; biblical studies (Hebrew Bible)

I confirm that I have read this submission and believe that I have an appropriate level of expertise to confirm that it is of an acceptable scientific standard, however I have significant reservations, as outlined above.

# Response

**Published:** 2026-02-05

**Authors:** Hanna Paulmann

**Affiliations:** Religions, University of Hamburg Faculty of Humanities, Hamburg, Hamburg, Germany

Dear Dr. Richard Pleijel,   thank you very much for taking the time and effort to review this paper. I appreaciate your thoughtful suggestions and the literature you recommended. For the new version, I have removed the discussion from table 4a and turned it back into the footnote that it was supposed to be. The narrow focus on Samuel and his notes in this paper is due to the state of the field of medieval Jewish philosophy. The interest in the philosophical manuscripts themselves as philosophical objects is a rather recent development and many features of these manuscripts, including, but not limited to marginal notes, have never been studied before. The HEPMASITE project, therefore, conducts case studies on those manuscripts to build the fundament for further, and broader research. In response to your suggestions, I have therefore added the aim of the project to the introduction to make clear how this paper fits into the project and mentioned the fields that can profit from its results (history of Jewish philosophy and translation studies). In the conclusion, I have engaged with the concepts of “agency” and “voice” in reference to the papers you recommended, which helped me to stress the importance of taking translators seriously because of their agency and the importance of studying paracontent to discern their voices. Further, I have pointed out that the results of my paper, and similar research dedicated to translation practices in Hebrew philosophical manuscripts, should be taken into consideration by scholars of translator notes within the field of translation studies, which, so far, seems primarily concerned with modern, printed translations. Finally, I have removed the statement that Samuel was not translating mechanically and explained in section 3.3 that this problem might be relevant to the field of classics because it questions the authenticity of (parts of) the passage under discussion as well as its transmission history in the Greek context.   Best regards, Hanna Paulmann

# Reviewer report: Reviewer response for version 1

**DOI:** 10.21956/openreseurope.22236.r62080

**Published:** 2025-11-11

**Authors:** jacobus naude

**Affiliations:** 1 University of the Free State, Bloemfontein, South Africa

Review of “A Translator at Work: Samuel ben Judah of Marseilles’s Translation Notes on Alexander’s *De Anima*” for *Open Research Europe*

 2025 10-06

 In the Middle Ages, many classical Greek works were translated into Arabic and then subsequently translated into Hebrew. This article examines the notes of the translator Samuel ben Judah of Marseilles in his translation of the Arabic translation (now lost) of the Greek work *De Anima* by Alexander. The study considers the translator’s notes both from a philological (text-critical) and translation viewpoint. Of particular interest are the translator’s explicit descriptions of text critical problems as well as the translator’s intervention to solve those problems.

 The article makes an important contribution to the history of translation in the Middle Ages, especially in the very fruitful interchange between Arabic and Hebrew scholars. It creates new knowledge and should be indexed. At the same time, the study would be improved by conceptualising the translation issues involved through the lens of current translation studies, with reference to two particular recent theoretical developments.

 First, a translator’s notes are “paratexts” (alternatively called “metatexts”), which stand alongside the translation and are intended to shape the reader’s interpretation of the translated text. The author would do well to shape the  description of Samuel ben Judah’s “notes” as “paratexts”, which will provide further insight into the kinds of information conveyed and the means for conveying it. For an introduction, see Batchelor’s book *Translation and Paratexts*. The author could contribute to the discussion with respect to the text critical aspects of the paratextual notes in the texts described in this article.

 Second, recent theoretical advances by Kobus Marais in the semiotics of translation from the standpoint of complexity thinking view translation (and re-translation) as complex and emergent. His recent works include *Translation Theory and Development Studies: A Complexity Theory Approach* (2014), *A (Bio)Semiotic Theory of Translation: The Emergence of Social-Cultural Reality* (2018) and *Trajectories of Translation: The Thermodynamics of Semiosis* (June 2023) and an edited volume Translation Beyond Translation Studies (2022). He has also published edited volumes with Reine Meylaerts, namely *Complexity Thinking in Translation Studies: Methodological Considerations *(2018), *Exploring the Implications of Complexity Thinking for Translation Studies* (2021), *The Routledge Handbook of Translation Theory and Concepts *(2023) and *The complexity of social-cultural emergence: Biosemiotics, semiotics and translation studies* (2024, with Maude Gonne as second editor).

 Corrections:

 --Arabic transliteration throughout needs to be corrected in the published version – replacing of question mark with proper diacritic.

 --p. 5 col. 1, para 2, line 3 – The Hebrew transliteration should be *hinne* not *hine*

 --p. 7 col 1. para 2, lines 8-9 – The Hebrew sentence has the words in the incorrect order when it broke into two lines.

 p. 8 col. 1 para 2, line 6 – For clarity, add “feminine noun” to the sentence – “and thus refers to the feminine noun “form” …”

 p. 8 col. 2, par 2 and p. 9 col. 1, para 1 – The two translations “for form is not matter” and “form is not matter” are not really distinguished in English. Perhaps translate the Hebrew of the first one more literally as “for that which is form is not matter”?

If any, are all the source data and materials underlying the results available?

Yes

Is the work original in terms of material and argument?

Yes

Is the work clearly and cogently presented?

Yes

Is the argument persuasive and supported by evidence?

Yes

Does the research article contribute to the cultural, historical, social understanding of the field?

Yes

Does it sufficiently engage with relevant methodologies and secondary literature on the topic?

Partly

Reviewer Expertise:

Linguistics of Premodern Hebrew (Syntax (synchronic and diachronic), Semantics, Discourse Analysis, Sociolinguistics and Applied Linguistics) as well as alterity and orality in Religious Translation. My research seeks to understand alterity (“otherness”) of ancient culture and to preserve it while representing it intelligibly for modern users. In my current research I utilise complexity theory to integrate seemingly disparate foci (pre-modern Hebrew linguistics and religious translation).

I confirm that I have read this submission and believe that I have an appropriate level of expertise to confirm that it is of an acceptable scientific standard, however I have significant reservations, as outlined above.

# Response

**Published:** 2026-02-05

**Authors:** Hanna Paulmann

**Affiliations:** Religions, University of Hamburg Faculty of Humanities, Hamburg, Hamburg, Germany

Dear Prof. Dr. Jacobus Naudé,   thank you very much for taking the time and effort to review this paper. I appreciate your thoughtful suggestions and the literature you recommended. In this new version of the paper, I have introduced Samuel’s notes as paracontent and referred to their function as a “threshold” to a text with reference to the book by Batchelor. I use the word “paracontent” instead of “paratext” or “metatext” in accordance with the terminology proposed by the Centre for the Study of Manuscript Cultures at the University of Hamburg. The thing my paper primarily contributes to the discussion on paracontent in translation studies is to offer further research approaches by paying special attention to the text-critical aspect of paracontent, which I added to the conclusion. The theoretical advances by Kobus Marais seem very relevant for anyone who studies translations as a cultural phenomenon and his commitment to re-introduce matter into the discussion of ideas is very much in line with the HEPMASITE project, in the context of which this paper was written.  However, to understand the particular ways in which Samuel was “constrained” and in what way his translation activity emerged from and contributed to the emerging philosophical culture in the Hebrew context, more research is needed, on the one hand in the form of case studies exploring individual translations, and on the other hand in the form of broader studies that investigate the development throughout the translation movement. Therefore, while it is important to be familiar with the complexity theory approach to translation, doing it justice would exceed the scope of this paper by far.   Finally, I thank you for your corrections that I have implemented into the new version. The Arabic transliteration was correct in the manuscript and in the proof and I do not know why they were not correctly presented in the published version of this paper. I have fixed them, but might have to contact the publisher after publication, if that does not solve the problem. The incorrect word order on p. 7 col 1. para 2, lines 8-9 has not been fixed after I remarked it in the proof, this, too, is something I need to contact the publisher about.  Regarding the distinction of the two phrases “for form is not matter” and “and form is not matter”, I have decided not to change my translation, because I want to convey that the change lies only in the first word of the phrase (from “for” to “and”). However, to make the difference more noticeable, they appear now in Italics and underlined.   Best regards, Hanna Paulmann

# Reviewer report: Reviewer response for version 1

**DOI:** 10.21956/openreseurope.22236.r62585

**Published:** 2025-11-08

**Authors:** Yehuda Halper

**Affiliations:** 1 Bar Ilan University, Ramat Gan, Israel

This is an important contribution to an area of study that is until now almost entirely neglected. I refer to treating the marginalia of a translator as their own contribution to a study. The only work to do this has been Carlos Frankel's study of Samuel Ibn Tibbon's marginal notes (see note 9). Still, this is a very different work, since it is marginalia to a scientific text by a single author, Samuel ben Judah of Marseilles. There has been an important study of this thinker by Berman (see note 6), but this new study adds a great deal of nuance that is not present in the earlier study.

 There are three things that I would suggest for improvement. 

 1. The movement between the notes and the manuscripts is cumbersome. The folio listed is usually following Paris BNF 893, but not always (in the first one). The folio numbers of each manuscript in the edited note should be mentioned each time. 

 2. How do we know that these notes are all by Samuel ben Judah? Some of them are attributed to him in some (or all?) manuscripts. Note 1 is not attributed, though I could not find it in the manuscript. But these could be misattributions made at an earlier ms source. This should be addressed in detail and explained. Reference here should be made to Fraenkel's work, which notes the difficulty in attributing to Samuel Ibn Tibbon every marginal note conveyed in his name.

 3. In 3.1.Note 1, the author notes that the Greek πρότερον comes into Hebrew as יקראו. It should be noted that in many medieval Hebrew scripts יקראו is quite similar to יקדמו and that the latter may have been a reading earlier in the chain of transmission. ידמו is, in fact, a fairly good translation of πρότερον.

If any, are all the source data and materials underlying the results available?

Yes

Is the work original in terms of material and argument?

Yes

Is the work clearly and cogently presented?

Yes

Is the argument persuasive and supported by evidence?

Yes

Does the research article contribute to the cultural, historical, social understanding of the field?

Yes

Does it sufficiently engage with relevant methodologies and secondary literature on the topic?

Yes

Reviewer Expertise:

Medieval Jewish and Islamic Philosophy. Transmission of Greek philosophy into Hebrew and Arabic. Medieval Hebrew manuscripts.

I confirm that I have read this submission and believe that I have an appropriate level of expertise to confirm that it is of an acceptable scientific standard.

# Response

**Published:** 2026-02-05

**Authors:** Hanna Paulmann

**Affiliations:** Religions, University of Hamburg Faculty of Humanities, Hamburg, Hamburg, Germany

Dear Prof. Dr. Yehuda Halper,   Thank you very much for taking the time and effort to review this paper and for your thoughtful suggestions. In this new version, I have (1.) added the folio numbers of all manuscripts to the tables in the paper itself and the extended data. Regarding (2.) the authorship of the notes, all of them are attributed to “Samuel the translator” in at least one manuscript. There are, in fact, only two instances in Paris 894 in which that is not the case. Note 9 is attributed to “Samuel” without the clarification that he is the translator and note 11 is not attributed at all there. Note 1 is attributed to “Samule the translator” in the manuscripts in which it appears (Paris 894 and Munich 389), but this note is wholly missing from Paris 893. While it is true that we do not have any indubitable proof that Samuel himself authored those notes, there is no evidence that they were not composed by Samuel. To the contrary, not only are they attributed to him, but they are also coherent with what he described in the colophon and many of them refer to the translation process and the Arabic manuscript that served as the source for the translation. The burden of proof, therefore, would lie with one who claims that they are not by Samuel. Finally, your suggestion that (3.) the Greek πρότερον might have been translated as יקדמו, which was later corrupted to יקראו would be very plausible if the note was not written by Samuel. Since Samuel, however, translated the text from the Arabic, and notices the problem with that phrase, the problem must have already been present in the Arabic source. The fact the note 1 discusses alternative translations supports my view that this note is indeed authored by Samuel himself and that the problem did not arise in the transmission of the Hebrew translation.   Best regards, Hanna Paulmann
